# Annotations

**Published:** 1908-05-02

**Authors:** 


### ANNOTATIONS.

### Electro-Therapeutics.

Recent developments in the medical application of electricity have not yet gained the full attention of the profession, but it is satisfactory to observe that efforts are now being made to obtain a fuller notice of advances in a department of practice which has too long been treated with neglect and even with suspicion. One of the most significant movements in this respect was to be traced in the announcement that the Royal Society of Medicine, on its establishment, was to include an electro-therapeutic section, and further evidence in the same direction may be found in the intimation that this section is about to hold one of its ordinary meetings, not in London, but in Glasgow. The programme for this meeting is framed on a decidedly ambitious scale. It includes the following papers: "Interrupted Currents for Electrical Testing and Treatment," by Dr. H. Lewis Jones; "Some Reflections based upon the Work done in the Electrical Department of the Royal Infirmary, Edinburgh," by Dr. Dawson Turner; and "Ionic Medication in the Treatment of some Obstinate Cases of Pelvic Disease in Women," by Dr. Samuel Sloan. In addition, there is to be an exhibition on a large scale of electrical apparatus, and in this most of the leading manufacturers will take part; visits will also be paid to the electrical installations at the Royal and Western Infirmaries. The date of the meeting is May 22, and the Chairman Mr. William Deane Butcher; Dr. W. F. Somerville is Honorary Secretary, and upon him the main responsibility for organising the local arrangements has fallen. The electro-therapeutic section is certainly to be congratulated on the enterprise and enthusiasm of its officers. They have afforded an example which may well be imitated by other sections, and which contradicts the timid fear that the formation of the Royal Society of Medicine will mean the paralysis of all initiative as a result of the restraining influence of the central organisation. The Glasgow meeting is to be open to all medical practitioners, and its success is practically assured.

### Doctors' Bills.

*Truth* inquires why it is that doctors alone of professional men send in bills at intervals of six months or a year without any statement of items. "We all recognise," continues the Editor, "that our doctors would not consciously ask us to pay what was not due to them; but they are no more secure against mistakes in making out accounts than the butcher, the baker, or the candlestick maker, especially when the business is left in the hands of a bookkeeper." This question is not a new one; it has been asked, and answered more or less satisfactorily at intervals during the last half-century or longer. But when it is repeated in the editorial columns of an influential lay paper, and is there publicly discussed in a temperate and reasonable manner, some public explanation or justification on the part of the medical profession is called for. And yet it must be confessed that the answer to this simple question is not easy to give in straightforward language, and, when given, it is not altogether satisfactory and convincing. To say that it is the accepted time-honoured custom of an honourable and learned profession to send out half-yearly or yearly accounts in the form of a lump sum "for professional attendance" does not really explain matters. Nor is it an adequate reply to say that the great majority of patients are content to accept this method without question or comment. It is, moreover, difficult to maintain that the dignity of the profession precludes a medical man from rendering a detailed statement of his services; since in the sister profession of the law accounts are rendered with scrupulous minuteness. The relations between doctor and patient may be more intimate and sacred than those between solicitor and client; health and life may be more important than litigation and worldly affairs; but, even so, one cannot deny that the difference is only one of degree, and that in each case there is a business relationship which demands a business-like financial attitude. It is perfectly true that the patient, if he wishes for a detailed statement of the medical services rendered and the prices charged, has only to demand an inspection of his doctor's books; but this is a course which most people hesitate to adopt, since it implies some degree of distrust, and even of ingratitude. The acquiescence of the public in this practice would, therefore, seem to be more in the nature of a tribute to our profession than a valid argument in favour of itemless bills.

### Death of Professor von Schrötter.

The proceedings of the International Laryngological Congress at Vienna last week were interrupted by a tragic occurrence, which cast a gloom over the entire assembly. Professor Leopold Schrötter von Kristelli, the distinguished Austrian laryngologist, who had delivered an inaugural address on the afternoon of Tuesday, April 21, in his capacity as honorary President of the Congress, died suddenly of heart failure during the course of the same evening. Professor von Schrötter was in his seventy-first year, and was justly famous beyond the limits of the medical profession for his valuable contribution towards the progress of laryngology, and for his large share in the international movement for the prevention of tuberculosis. Following the lines laid down by Türck and Czermak—whose achievements he celebrated in his address on the day of his death—he played for many years a prominent part in the development of laryngological science. He was the founder of the Alland Sanatorium for Tuberculosis, near Vienna. In 1887 he was summoned to San Remo to the German Crown Prince, afterwards Emperor Frederick, who was suffering from malignant disease of the larynx. Owing to the widespread public interest which was taken in this case, Professor von Schrötter's name became familiar throughout Europe. The news of his death was received with deep regret by the assembled delegates. Owing to the wishes of his family, the Congress did not adjourn, but a deputation, including Sir Felix Semon, was sent to offer the condolences of the delegates.

